# Does Japan’s national nutrient-based dietary guideline improve lifestyle-related disease outcomes? A retrospective observational cross-sectional study

**DOI:** 10.1371/journal.pone.0224042

**Published:** 2019-10-17

**Authors:** Daisuke Yoneoka, Shuhei Nomura, Kayo Kurotani, Shiori Tanaka, Keiji Nakamura, Hisayuki Uneyama, Naoki Hayashi, Kenji Shibuya

**Affiliations:** 1 Division of Biostatistics and Bioinformatics, Graduate School of Public Health, St. Luke’s International University, Tokyo, Japan; 2 Department of Global Health Policy, Graduate School of Medicine, The University of Tokyo, Bunkyo-ku, Tokyo, Japan; 3 Department of Nutritional Epidemiology and Shokuiku, National Institute of Health and Nutrition, National Institutes of Biomedical Innovation, Health and Nutrition, Shinjyuku-ku, Tokyo, Japan; 4 Epidemiology and Prevention Group, Center for Public Health Sciences, National Cancer Center, Chuo-ku, Tokyo, Japan; 5 Ajinomoto Co., Inc., Chuo-ku, Tokyo, Japan; International University of Health and Welfare, School of Medicine, JAPAN

## Abstract

**Objective:**

The Japanese government has developed and promoted a national nutrient-based dietary guideline for preventing lifestyle-related chronic disease. However, its impact in a real-life setting has never been evaluated. We performed a critical appraisal of the guideline by examining the association between adherence to the guideline and lifestyle-related outcome indicators.

**Methods:**

This is a retrospective observational cross-sectional study using nationally representative data on health and nutrition characteristics from the 2016 National Health and Nutrition Survey of Japan. We considered 3,861 participants aged ≥20 years, with evidence of low health risks of diabetes, hypertension, hyperlipidemia, and obesity. Five health outcome indicators (hemoglobin A1c (HbA1c), systolic blood pressure, diastolic blood pressure, high-density lipoprotein cholesterol, and body mass index), were employed. A summary score was developed to reflect adherence to the recommended intake of seven nutrients defined by the guideline, including proteins, fat, saturated fatty acid, carbohydrate, dietary fiber, sodium, and potassium. Multivariate quartile regression approaches were employed to examine the association between the adherence score and the health outcome indicators, adjusting for the covariates.

**Results:**

Overall, the proportion of those who adhered to the guideline (adherence rate) for all seven nutrients was only 0.3%. There was considerable variation in the adherence rate between the different nutrients, from 24.2% to 61.8%. After adjustment for covariates, in most health outcome indicators, regardless of age category and quartile, there was no clear association between the guideline adherence score and indicators.

**Conclusions:**

There is plenty of scope for improving the guideline. Nutrient impact on health may not necessarily depend on the amount of each nutrient in the diet. The significance and contribution of synergies between nutrients and complex interactions within foods to health outcomes need to be explored in future guideline updates.

## Introduction

Japan is one of the most successful countries in the world on account of excellent health outcomes [1]. The decline in disease burden in Japan in the last decades is largely due to a substantial decline in the incidence of major lifestyle diseases (e.g. cerebrovascular diseases, ischemic heart diseases, and some cancers) [2]. However, today, these diseases still remain top causes of mortality and morbidity, and the progress in population health has slowed down primarily due to the levelling off of the decline in disease burden. More efforts are therefore needed, to tackle major risk factor [2].

According to Nomura and colleagues (2017), behavioral risk factors made a greater contribution to the overall disease burden, which included mortality and morbidity (25.2%) in 2015 than metabolic risks (16.4%), or environmental and occupational risks (4.4%) [2]. Unhealthy diets (high in sodium or low in whole grains, fruits, vegetables, and nuts and seeds) are an important modifiable behavioral risk factor of many health conditions in both men and women in Japan, accounting for, respectively, 13.8% and 9.3% of disease burden in 2015 [2]. A recent rising tide of evidence and growing interest in healthy diet, driven by the commercialization of healthy foods and food ingredients, highlights the substantial opportunities for a healthier population in Japan through healthy diet [2–4].

The Japan Ministry of Health, Labour and Welfare (MHLW) established the Dietary Reference Intakes (DRIs) for Japan in 2005 and revised it in 2010 and 2015 [5], which is expected to be revised again in 2020. The DRIs is a unique national nutrient-based dietary guideline in Japan, developed on the basis of the Health Promotion Act and promoted by the government. The guideline promotes healthy diet by avoiding adverse health effects due to inadequacy and excessive nutrient intakes, and for preventing lifestyle-related chronic disease. DRIs include tentative dietary goals (TDGs), defined as the intake range for seven nutrients that Japanese people should aim for, to reduce the risk of lifestyle-related chronic diseases (details are presented below). Consumption that is in excess or falls short of the TDGs potentially confers an increased risk of chronic diseases.

The DRIs/TDGs guidelines are widely applied in practice, including in commercial food production and nutritional education [6, 7]. For example, the Japanese Food Guide Spinning Top was developed based on DRIs, which is a chart designed for the general public, indicating with illustrations, the recommended daily servings for some food group [8, 9]. However, despite convincing evidence that nutrients are essential for human health, to our knowledge, the impact of TDGs on health outcomes has never been evaluated using nationally representative data. The objective of the present study was to examine the association in terms of direction, magnitude, and significance between the adherence to the TDGs and health outcomes (determined based on the availability of J-NHNS data) using the National Health and Nutrition Survey of Japan (J-NHNS).

## Materials and methods

### Settings

#### Japan National Health and Nutrition Survey

We used data from the National Health and Nutrition Survey of Japan (J-NHNS) performed in 2016. J-NHNS is a cross-sectional household survey, carried out once a year in November by the MHLW [10, 11]. The objective of the annual J-NHNS is to assess the physical status, nutritional intake, and lifestyle of the Japanese people, for comprehensive promotion of the population health. The J-NHNS comprises of the following three parts: 1) physical examination including a blood test, performed by physicians at designated community centers; 2) an in-person dietary survey of a weighted single-day dietary record of households, with proportional distribution (of the meal pattern, food items, etc.) within the house, conducted by registered dietitians who visits each household and checks the completeness of recording forms; and 3) a self-reported lifestyle questionnaire (including occupation, smoking, and alcohol consumption), administered along with the dietary survey. The intake of energy and nutrients were estimated (as part of J-NHNS) based on the dietary record and the corresponding food composition list in the Standard Tables of Food Composition in Japan (sixth revised edition as at 2016) [12]. Other data included age (as of 1 November 2016) and sex.

In the 2016 J-NHNS, using a stratified single-stage cluster sample design, census enumeration areas were drawn from each prefecture (the country's first-order administrative division) to obtain a nationally representative sample of the non-institutionalized Japanese population. A total of 24,187 households from 475 randomly selected census enumeration areas were eligible for the survey. The response rate of households was 44.4% (10,745 households; 26,354 individuals aged 1 year and older) [13].

#### Five outcomes of interest

Given the availability of J-NHNS data, we considered the following five health outcome indicators related to nutrients: hemoglobin A1c (HbA1c), systolic blood pressure (SBP), diastolic blood pressure (DBP), high-density lipoprotein cholesterol (HDL-C), and body mass index (BMI). They are known to be associated with lifestyle-related chronic diseases (diabetes, high blood pressure, hyperlipidemia, obesity, etc.); with higher values indicating a greater risk of the diseases (except for HDL-C, where the lower the value, the greater the risk of diseases). HbA1c was measured in National Glycohemoglobin Standardization Program (NGSP) units.

#### Eligible participants

Given the nature of the cross-sectional study design of J-NHNS, reverse causation (also called reverse causality) is possible and cannot be ruled out (discussed in the Discussion section) [14, 15]. Thus, to minimize the likelihood of reverse causation as much as possible using the available J-NHNS data, we excluded those who might be aware (or ought to be aware) of their health risks including: those with a HbA1c of ≥6.5% or taking antihyperglycemic agents for diabetes; those with an LDL-C of ≥140 mg/dL or HDL-C of ≤40 mg/dL, or taking antihyperlipidemic agents; those with a SBP of ≥140 mmHg or DBP of ≥90 mmHg, or taking antihypertensive agents; and those with a BMI of ≥25.

Furthermore, since the blood test and lifestyle questionnaire were not administered to children and the younger population aged less than 20 years, only those aged 20 years or older were considered. Because of the unique nutritional needs of pregnant women, we also excluded them from the analysis.

#### Dietary Reference Intakes for Japanese and seven nutrients of interest

DRIs proposes a desirable intake of energy and nutrients for Japanese people to maintain and promote their health [5]. Applicable populations are healthy individuals. TDGs—one of the nutritional indices included in DRIs with emphasis on lifestyle-related diseases, are highlighted among others: e.g. recommended dietary allowance, adequate intake, and tolerable upper intake level. This is because—as the DRIs claims—it is based on a sufficient scientific evidence to support the desired intake. In 2015, DRIs reported TDGs for seven nutrients (proteins, fat, saturated fatty acid, carbohydrates [excluding sugar], dietary fiber, sodium, and potassium) by age category (18–69 and ≥70 years old) and sex (Table 1), where there was sufficient evidence; these were considered in this study (i.e. target nutrients of interest). TDGs of proteins, fat, saturated fatty acid, and carbohydrate are expressed as a percentage of the total energy intake. This is because its goals, in reality, are not independent of other energy sources or of the total energy goals for the individual. Details, including calculation methods of TDGs for each nutrient, are described elsewhere [5].

**Table 1:** Tentative dietary goals of seven nutrients included in the study (MHLW 2015) [5].

	Women	Men
Proteins*		
18–69	13–20	13–20
70+	13–20	13–20
Fat*		
18–69	20–30	20–30
70+	20–30	20–30
Saturated fatty acid*		
18–69	≤7	≤7
70+	≤7	≤7
Carbohydrate*		
18–69	50–65	50–65
70+	50–65	50–65
Dietary fiber [g/day]		
18–69	≥18	≥20
70+	≥17	≥19
Sodium**		
18–69	≤7	≤8
70+	≤7	≤8
Potassium [mg/day]		
18–69	≥2,600	≥3,000
70+	≥2,600	≥3,000

* percentage in total energy intake

** salt equivalent values [g/day]: sodium [mg] x 2.54/1,000.

### Data analysis

#### Adherence score to TDGs

Globally there are more than 25 indices measuring overall diet quality and/or variety that mostly assesses adherence to particular dietary recommendations or guidelines, specific to the country where the indices were developed [16]. In Japan, Oba et al. (2009) developed a dietary adherence index for the Japanese population [9], and identified, using the index, significant (for women) and non-significant (for men) associations of higher adherence to the Japanese Food Guide Spinning Top (consisting of five basic food [not nutrition] categories) with lower all-cause mortality [9].

According to their methodology, we calculated a summary score, which reflects adherence to TDGs from a maximum of 10 to a minimum of 0 for each nutrient and each individual; with a higher score indicating closer adherence and thus better diet. If individuals consumed the TDGs of a nutrient of interest, 10 points were given. If individuals exceeded or fell short of the TDGs, the score was calculated proportionately between 0 and 10: if an individual consumed less than the TDGs, the score was calculated with the following formula:

$$10\times \frac{amount\;of\;nutrition\;intake}{lower\;limit\;of\;TDGs}$$

while if an individual consumed more than the TDGs, the score was calculated with the following formula:

$$10-10\times \frac{amount\;of\;nutrition\;intake-upper\;limit\;of\;TDGs}{upper\;limit\;of\;TDGs}$$

Scores for seven nutrients of interest were summed to obtain a total score ranging from 0 (the lowest adherence) to 70 (the highest adherence). Unless indicated otherwise, we present adherence score in terms of the total score for the seven nutrients.

### Statistical analysis

Differences in values between the stratified groups (according to adherence score quantile or BMI) were tested using Chi-squared, Fisher’s exact, or ANOVA tests depending on the nature of the data. Then the analysis proceeded by age category (20–39, 40–59, or ≥60 years old) using multivariate regression approaches to quantify the association of adherence score with the health outcome indicators, adjusting for the following covariates: sex, occupation (engaged in primary, secondary, tertiary, or other industries), smoking status (current, former, or never smoke), and alcohol consumption status (frequently [≥3 days/week], sometimes [≤3 days/week], former, or never drink). To adjust for possible non-linearity of associations between outcome indicators and total energy intake, we considered restricted cubic spline with three knots placed at the 25th, 50th, and 75th percentiles of total energy intake as a covariate in the following regression models.

Two regression models were used in this study: (1) a conventional linear regression, and (2) a linear quantitative regression. First, to estimate the conditional mean of the health outcome indicators as a function of the adherence score with adjustment for covariates, we applied a conventional linear mixed effect regression approach, which included a random intercept for the household, to control for correlation in the data of individuals residing in the same household. Second, a linear quantile regression approach with the random intercept for the household effect was employed to estimate the conditional quantile function of the adherence score, adjusting for covariates. A quantile regression, a statistical tool, extends the conventional mean regression method beyond its application to characterize the entire conditional distribution of the outcome variable (i.e. health outcome indicators). It also provides more robust results with deviations from normality of the residual [17]. Therefore, a quantile regression allows for the examination of any distribution of the outcome variable, and modelling all the parameters (location, scale, and shape) of the distribution, as functions of the explanatory variables (i.e. adherence score). In the present study, the estimation was performed independently for each quartile (25th, 50th, and 75th percentiles). The optimization algorithm for regression estimation relies on the asymptotic Laplace-based likelihood approximation using Gauss-Hermite quadrature with seven knots [18].

It should be noted that BMI is a key physiological predictor of total energy intake while the TDGs of proteins, fat, saturated fatty acids, and carbohydrates are not mutually exclusive, because they are expressed as a percentage of the total energy intake. Therefore, with the sensitivity analyses, the regression models were also estimated by the two different BMI categories to determine differences in the function of the adherence score at different BMI levels. According to the DRIs, the recommended healthy BMI ranges were 18.5 to 25.0 (18–49 years old), 20.0 to 25.0 (50–69 years old), and 21.5 to 25.0 (70 years old or older) for both sexes; which were therefore regarded as appropriate categories. BMI, which falls below these ranges were considered as a ‘poor’ category while the remaining were classed as the ‘proper’ category. Finally, other sensitivity analyses included the regression models with no eligibility criteria for participants. We considered p-values less than 0.05 as statistically significant.

### Ethics approval

Ethical approval for the study was granted by the ethics committee of The University of Tokyo (11964). Written informed consent was not required, as this study was a secondary analysis of anonymized data that is collected routinely by the MHLW.

## Results

A total of 3,861 participants were eligible and their data were analyzed in this study. Overall, the proportion of those who adhered to guideline (adherence rate to TDGs) for all seven nutrients was only 0.3%, while the highest adherence rate was observed with proteins (64.8%), followed by carbohydrate (61.8%), fat (50.0%), saturated fatty acid (46.3%), sodium (27.6%), potassium (27.5%), and dietary fiber (24.2%).

The mean and median adherence scores to TDGs were 60.03 (standard deviation [SD] 6.37) and 60.70 (interquartile range [IQR] 56.30–64.90). Scores ranged from 24.9 to 70.0. Compared to those with a lower adherence score, those with a higher score were older and more likely to be women, be engaged in primary industry, and have a higher total energy intake; and were less likely to be current smokers or to drink alcohol frequently (≥3 days/week) (Table 2).

**Table 2:** Basic characteristics of the study population by quantile adherence score.

	1Q [24.9–57.3]	2Q [56.3–60.7]	3Q [60.7–64.9]	4Q [64.9–70.0]	p-value*
Sample size	966	965	965	965	
Mean age (SD)	44.8 (15.0)	49.3 (15.2)	52.3 (16.1)	57.3 (15.1)	<0.001
Sex (%)					0.072
Women	550 (56.9)	595 (61.6)	664 (68.7)	847 (87.7)	
Men	416 (43.1)	370 (38.3)	301 (31.2)	118 (12.2)	
Occupation (%)					<0.001
Primary	28 (2.9)	38 (3.9)	42 (4.3)	33 (3.4)	
Secondary	580 (60.0)	553 (57.2)	484 (50.1)	373 (38.6)	
Tertiary	133 (13.8)	119 (12.3)	104 (10.8)	96 (9.9)	
Other	225 (23.3)	255 (26.4)	335 (34.7)	463 (47.9)	
Smoke (%)					<0.001
Current	240 (24.8)	158 (16.4)	97 (10.0)	32 (3.3)	
Former	90 (9.3)	97 (10.0)	91 (9.4)	53 (5.5)	
Never	635 (65.7)	707 (73.2)	777 (80.4)	878 (90.9)	
Alcohol (%)					<0.001
Frequently (≥3days/week)	283 (29.3)	293 (30.3)	247 (25.6)	157 (16.3)	
Sometimes (<3days/week)	382 (39.5)	330 (34.2)	356 (36.9)	377 (39.0)	
Former	16 (1.7)	17 (1.8)	15 (1.6)	16 (1.7)	
Never	282 (29.2)	321 (33.2)	346 (35.8)	412 (42.7)	
Mean HbA1c [%] (SD)	5.4 (0.3)	5.5 (0.3)	5.5 (0.3)	5.6 (0.3)	<0.001
Mean SBP [mmHg] (SD)	116.0 (11.6)	117.0 (11.7)	116.9 (12.1)	118.1 (12.0)	<0.01
Mean DBP [mmHg] (SD)	72.7 (8.4)	73.2 (8.1)	73.2 (8.3)	72.9 (8.0)	0.421
Mean HDL-C [mg/dL] (SD)	66.7 (15.5)	67.2 (15.5)	67.7 (14.9)	69.4 (15.2)	<0.001
Mean BMI (SD)	21.1 (2.1)	21.0 (2.2)	20.9 (2.1)	20.9 (2.2)	0.351

Q: quantile of the score; SD: standard deviation; HbA1c: hemoglobin A1c; SBP: systolic blood pressure; DBP: diastolic blood pressure; HDL-C: high-density lipoprotein cholesterol; BMI: body mass index

* Chi-squared tests, Fisher’s exact tests, or ANOVA tests to compare the differences in the distributions and proportions of the characteristics across the quantiles (1Q, 2Q, 3Q, and 4Q), depending on the nature of the data.

Intakes of the seven nutrients among the study population are presented by quantile adherence scores in Table 3. The groups with higher adherence scores had a greater adherence rate for each nutrient (p<0.001), except for sodium, with an opposing trend; the greater adherence rate was observed in the group with a smaller adherence score (p<0.001). Intakes of the seven nutrients by BMI category are shown in Table 4. The proper BMI category did not necessarily have a higher adherence rate for each nutrient, although it had the lowest total energy (<0.001).

**Table 3:** Nutrient intake of the study population by quantile adherence score.

Score	1Q [24.9–57.3]	2Q [56.3–60.7]	3Q [60.7–64.9]	4Q [64.9–70.0]	p-value*
Mean total energy [kcal/day] (SD)	1766.0 (631.2)	1846.8 (564.1)	1909.9 (496.9)	1939.8 (399.2)	<0.001
Proteins**					
Mean (%) (SD)	13.9 (3.7)	14.3 (2.8)	15.0 (2.8)	15.6 (2.6)	<0.001
Adherence rate^†^ (%)	48.9	62.2	69.8	78.1	<0.001
Fat**					
Mean (%) (SD)	31.3 (10.0)	26.7 (7.0)	26.8 (5.8)	25.2 (4.5)	<0.001
Adherence rate^†^ (%)	22.3	49.5	55.8	72.4	<0.001
Saturated fatty acid**					
Mean (%) (SD)	9.2 (3.7)	7.3 (2.6)	7.1 (2.1)	6.4 (1.5)	<0.001
Adherence rate^†^ (%)	26.2	46.6	48.3	63.9	<0.001
Carbohydrate**					
Mean (%) (SD)	50.7 (11.6)	55.3 (8.1)	55.5 (7.1)	57.8 (5.8)	<0.001
Adherence rate^†^ (%)	35.1	61.6	68.6	80.5	<0.001
Dietary fiber [g/day]					
Mean (SD)	10.1 (5.2)	13.1 (6.2)	15.7 (5.2)	20.2 (5.6)	<0.001
Adherence rate^†^ (%)	4.0	12.2	22.5	58.0	<0.001
Sodium [mg/day]					
Mean (SD)	3554.3 (1622.8)	3739.7 (1602.5)	3764.7 (1226.2)	4016.6 (1331.9)	<0.001
Adherence rate^†^ (%)	37.8	31.1	24.8	16.9	<0.001
Potassium [mg/day]					
Mean (SD)	1683 (780.6)	2067.6 (859.9)	2429.3 (723.5)	2943.4 (671.9)	<0.001
Adherence rate^†^ (%)	6.1	13.9	27.2	62.7	<0.001

Q: quantile of the score; SD: standard deviation

* Chi-squared tests, Fisher’s exact tests, or ANOVA tests to compare the differences in the distributions and proportions of the characteristics across the quantiles, depending on the nature of the data

** percentage in total energy intake

†percentage of those adhered to TDGs.

**Table 4:** Nutrient intake of the study population by BMI.

BMI	Poor^#^	Proper^#^	p-value*
Mean total energy [kcal/day] (SD)	61.3 (6.1)	59.6 (6.4)	<0.001
Proteins**			
Mean (%) (SD)	15.1 (3.1)	14.5 (3.1)	<0.001
Adherence rate^†^ (%)	67.0	64.0	0.188
Fat**			
Mean (%) (SD)	26.8 (7.3)	27.8 (7.5)	<0.001
Adherence rate^†^ (%)	51.4	49.5	0.566
Saturated fatty acid**			
Mean (%) (SD)	7.3 (2.7)	7.6 (2.8)	<0.01
Adherence rate^†^ (%)	48.8	45.4	0.061
Carbohydrate**			
Mean (%) (SD)	55.6 (8.5)	54.6 (8.9)	<0.01
Adherence rate^†^ (%)	64.8	60.3	<0.05
Dietary fiber [g/day]			
Mean (SD)	15.7 (7.1)	14.5 (6.5)	<0.001
Adherence rate^†^ (%)	30.4	22.0	<0.001
Sodium [mg/day]			
Mean (SD)	3715.4 (1485.6)	3787.4 (1457.1)	0.182
Adherence rate^†^ (%)	28.2	27.5	0.721
Potassium [mg/day]			
Mean (SD)	2398.6 (934.9)	2239.6 (873.6)	<0.001
Adherence rate^†^ (%)	34.0	25.2	<0.001

BMI: body mass index; Q: quantile of the score; SD: standard deviation

* Chi-squared, Fisher, or Student's T tests, depending on the nature of the data

** percentage in total energy intake

† percentage of those adhered to TDGs:

^#^ poor: <18.5 for 18–49 years old, <20.0 for 50–69 years old, <21.5 for 70 years old or more; proper: 18.5–24.9 for 18–49 years old, 20.0–24.9 for 50–69 years old, 21.5–24.9 for 70 years old or more

Table 5 shows the estimated regression coefficient of the adherence score to the health outcome indicators using linear mixed effect conventional and quantile regressions. After adjustment for covariates (sex, occupation, smoking status, alcohol consumption status, and total energy intake as restricted cubic spline), the null (non-significant) results were found for most outcome indicators regardless of age category and quartile. Thus, this indicates no clear association of TDGs adherence score (comprising the seven nutrients) with the health outcome indicators, considered in this study. SBP, among a group of 20–30 and 40–59-year-old participants, demonstrated significant results with positive regression coefficients for every quantile. Importantly, a positive coefficient means that the higher the adherence score, the greater the SBP, while better (lower) SBP levels indicate better overall control and a lesser risk of hypertension. At the first quantile for HDL-C among a group of 20–39 and 40–59-year–old participants, a significantly negative coefficient was estimated. This implies that the higher the adherence score, the lower the HDL-C, and an increased risk of hyperlipidemia.

**Table 5:** Estimated coefficients of adherence score using quantile/conventional regression: (A) for score quantile of 25% and 50%, (B) for 75% and mean.

(A)				
Score quantile	25%		50% (median)	
Age category	Coefficient (95% CI)	p-value	Coefficient (95% CI)	p-value
HbA1c [%]				
20–39	0.000 (-0.002 to 0.003)	0.699	0.003 (0.001 to 0.005)	<0.05
40–59	0.002 (-0.001 to 0.005)	0.178	0.004 (0.001 to 0.007)	<0.01
≥60	0.000 (-0.004 to 0.004)	0.899	0.002 (-0.002 to 0.006)	0.253
SBP [mmHg]				
20–39	0.003 (0.001 to 0.005)	<0.05	0.005 (0.003 to 0.008)	<0.001
40–59	0.004 (0.001 to 0.007)	<0.01	0.007 (0.003 to 0.010)	<0.001
≥60	0.002 (-0.002 to 0.006)	0.253	0.004 (0.000 to 0.009)	0.055
DBP [mmHg]				
20–39	0.000 (-0.096 to 0.096)	1.000	0.060 (-0.038 to 0.159)	0.222
40–59	-0.016 (-0.090 to 0.058)	0.667	0.047 (-0.024 to 0.118)	0.191
≥60	-0.082 (-0.163 to -0.001)	<0.05	-0.007 (-0.085 to 0.072)	0.866
HDL-C [mg/dL]				
20–39	-0.170 (-0.299 to -0.040)	<0.05	-0.070 (-0.203 to 0.063)	0.298
40–59	-0.254 (-0.392 to -0.116)	<0.01	-0.133 (-0.268 to 0.002)	0.054
≥60	-0.172 (-0.372 to 0.027)	0.089	-0.037 (-0.237 to 0.163)	0.712
BMI				
20–39	-0.011 (-0.029 to 0.007)	0.212	0.009 (-0.009 to 0.027)	0.319
40–59	-0.034 (-0.052 to -0.015)	<0.01	-0.013 (-0.032 to 0.005)	0.149
≥60	-0.014 (-0.042 to 0.014)	0.314	0.004 (-0.023 to 0.032)	0.747
(B)				
Score quantile	75%		Mean	
Age category	Coefficient (95% CI)	p-value	Coefficient (95% CI)	p-value
HbA1c [%]				
20–39	0.005 (0.003 to 0.008)	<0.001	0.003 (0.000 to 0.005)	<0.05
40–59	0.007 (0.003 to 0.010)	<0.001	0.004 (0.002 to 0.007)	<0.01
≥60	0.004 (0.000 to 0.009)	0.055	0.002 (-0.002 to 0.006)	0.250
SBP [mmHg]				
20–39	0.003 (0.000 to 0.005)	<0.05	0.027 (-0.069 to 0.122)	0.589
40–59	0.004 (0.002 to 0.007)	<0.01	0.007 (-0.093 to 0.108)	0.886
≥60	0.002 (-0.002 to 0.006)	0.250	0.059 (-0.067 to 0.186)	0.359
DBP [mmHg]				
20–39	0.141 (0.044 to 0.239)	<0.01	0.062 (-0.017 to 0.141)	0.123
40–59	0.115 (0.038 to 0.193)	<0.01	0.041 (-0.029 to 0.110)	0.249
≥60	0.057 (-0.025 to 0.138)	0.168	-0.017 (-0.110 to 0.076)	0.719
HDL-C [mg/dL]				
20–39	0.088 (-0.043 to 0.219)	0.182	-0.046 (-0.173 to 0.081)	0.474
40–59	0.035 (-0.108 to 0.178)	0.626	-0.108 (-0.244 to 0.027)	0.117
≥60	0.083 (-0.122 to 0.289)	0.419	-0.004 (-0.180 to 0.171)	0.961
BMI				
20–39	0.030 (0.012 to 0.048)	<0.01	0.011 (-0.009 to 0.031)	0.299
40–59	0.007 (-0.011 to 0.025)	0.468	-0.014 (-0.033 to 0.004)	0.135
≥60	0.025 (-0.003 to 0.053)	0.074	0.004 (-0.023 to 0.030)	0.789

HbA1c: hemoglobin A1c; SBP: systolic blood pressure; DBP: diastolic blood pressure; HDL-C: high-density lipoprotein-cholesterol; BMI: body mass index; CI: Confidence interval; adjusted for sex, occupation, smoking status, alcohol consumption status, and total energy intake as restricted cubic spline. The 'mean' indicates the parameter estimates of the conventional linear regression, while '25%', '50%', and '75%' show the parameter estimates of the quantile regressions at the 25th, 50th, and 75th percentiles of the adherence score distribution, respectively.

With the sensitivity analyses, the regression coefficients of adherence score to the health outcome indicators were also estimated by two different BMI categories (S1 and S2 Tables for the poor and proper categories, respectively), which yielded similar null results for most outcome indicators regardless of the age category and quartile, after adjusting for covariates. Other sensitivity analyses also resulted in similar results (S3 Table) in terms of direction, magnitude, and significance, except for SBP in 25th and 50th quantiles.

## Discussion

To our knowledge, the present study is the first to perform a critical appraisal of the DRIs by examining the association between adherence to the TDGs and lifestyle-related chronic outcome indicators. Overall, only 0.3% of the study population adhered to TDGs for all seven nutrients studied; and there was a large range in the adherence rate between the different nutrients from 24.2% to 61.8%. We found that a higher adherence score was related to older age and female gender (Table 2). A similar age and gender tendency with regard to dietary guideline adherence was observed in the U.S. population [19]. These findings indicate the difficulty among most Japanese, especially the younger male population, in meeting the TDGs for the nutrients in their daily diet.

While there was a clear tendency that a group with higher adherence score had the greater adherence rate for each nutrient, a poor adherence rate for sodium was observed in a group with higher adherence score (Table 3). Furthermore, that some stratified groups showed significant associations between a higher adherence score and greater SBP and DBP levels (Table 5), could be attributed to higher salt intake. One possible mechanism for this finding includes the fact that adding more vegetables, fruits and/or fish to meals, which is widely acknowledged to be a part of a well-balanced diet, may actually increase the amount of seasoning, which then results in greater sodium intake. In fact, the annual report of the 2016 J-NHNS reported that older people (who were found to have a higher adherence score in this study), are more likely not only to eat vegetables and fish, but were also more likely to have higher salt intake [13].

The daily salt intake of Japanese is known to be approximately 9.2 gram for women and 10.8 gram for men in 2016 [13], and most of it may derive from seasonings (soy sauce, table salt, and miso), bread, noodles, and other processed foods [20, 21]. According to a 2018 study conducted by Takimoto et al., using the data of the 2012 J-NHNS [22], 82.1% of the Japanese consumed soy sauce on the day the survey was conducted, which was equivalent to an average intake of 2.2 gram of salt. Also, many Japanese used table salt and miso (81.4% and 47.1%, respectively), which were equivalent to 1.6 gram and 1.9 gram of salt intake on average, respectively [22]. In addition to these seasonings, about 1–3% of the people consumed processed seafood (depending on the product), which corresponded to about 1 gram of salt intake [22]. About 1.0% of the people ate instant Chinese noodles (dried by frying, seasoned), which was equivalent to 2.2 gram of salt intake [22]. These results imply that it is not the food itself but the way in which it is cooked or seasoned that ultimately influences sodium levels in the body. The source of sodium should be clearly considered when addressing sodium intake reduction.

Importantly, the findings of the multivariate regression analyses imply that TDG adherence based on the seven nutrients has limited beneficial impact (and an adverse effect on some stratified groups) on the indicators, after adjusting for covariates. These findings support existing nutrition science understandings that the level of each nutrient, independent of the source of the nutrients, may not necessarily explain healthy diet [23, 24]. Nutrition science is evolving today and experiencing a paradigm shift from focusing on isolated nutrients, deficiency diseases, and their prevention, towards foods, chronic diseases, and overall diet patterns [24]. Such a scientific shift has been driven by emerging recognition of the diverse, complex health effects of different foods and dietary patterns beyond individual nutrients [25]. There are increasing numbers of food-based dietary guidelines aimed at preventing lifestyle-related chronic diseases [24], which commonly recommend that people eat more minimally processed, bioactive rich foods (e.g. grains, fruits, and vegetables) and avoid ultra-processed foods rich in ingredients such as sugars, fats, and industrial additives such as trans-fat and sodium [25]. Many studies reported that a quality diet with greater adherence to the food guidelines led to a lower risk of mortality and morbidity [8, 9, 26–28].

Our study findings highlight that the scope for improving the DRIs/TDGs is wide. As Jacobs et al. (2009) have reported, foods are more than just a collection of nutrients they contain [23]. There are synergies between nutrients and complex interactions within foods that help explain associations with health outcomes [23, 29, 30]. This is referred to as the 'food matrix [23]. Therefore, the significance and contribution of nutrient interactions in a food matrix to health outcomes also need to be explored in future guideline updates [31, 32]. The aforementioned are also our next research subject. In addition, while TDGs for proteins, fat, and carbohydrates were expressed as a percentage of total energy intake, a desirable energy requirement was not considered in a TDG context. In other words, for example, a high intake of carbohydrates was not considered a concern. Therefore, the reference intake for total energy that could maintain or achieve a proper BMI level should be determined while taking into account energy consumption (i.e. energy balance), although this is difficult due to the possibility of a high variability between individuals.

## Limitation

Our analyses are subject to similar limitations as are described for other studies concerning dietary adherence [8, 9]. First, dietary intake was based on a weighted single-day dietary record, which might not represent reproducibility of dietary patterns in the long term. Also, the single-day data does not reflect seasonal variations in dietary patterns. Also, self-reporting in the dietary survey and lifestyle questionnaire renders the participants susceptible to social desirability response bias and recall bias due to subjectivity and poor memory. Unfortunately, there are no data available for testing the validity of their responses. In addition, relying on household representatives to record dietary intake on the survey may lead to biased estimates of the dietary intake of individual respondents, particularly for those who work and have lunch outside the home on weekdays [11]. However, it should be noted that monitoring the daily dietary intake of individuals is challenging owing to the technical, financial, and practical difficulties, as well as barriers involving privacy issues that make people reluctant to participate in such monitoring exercises. In this context, we would like to emphasize that the nationally representative, large-scale data from J-NHNS offered a unique and valuable opportunity for us to perform this study with sufficient quality dietary data in order to better understand associations of adherence to TDGs with chronic diseases indicators.

Second, we could not rule out the confounding effects of unmeasured predictors of negative health outcomes, such as low physical activity and functional decline including frailty and sarcopenia among older adults [33]. Although J-NHNS recording forms include physical activity levels, most participants did not report it; thus, we were not able to adjust for potentially different physical activity levels among the study population in the analyses. Other social determinants of health including social, economic, and environmental conditions were also not considered in the study. There is the possibility that the effects of these unmeasured factors might conceal those of TDG adherence on the health outcome indicators.

Third, the different nutrients have not been weighted for adherence score calculation. Adherence to TDGs for each nutrient was scored from a maximum of 10 to a minimum of 0 for each individual, and scores for the seven nutrients were simply summed up to obtain the total score ranging from 0 (the lowest adherence) to 70 (the highest adherence). How the "best" adherence score (that is, the "best" weighting scheme) is actually defined requires a clear understanding of what the optimal weight is and how it could be optimized. This is a large study subject in itself [34, 35], and was beyond the scope of this study, so it shall be considered as the next research subject.

Fourth, the cross-sectional nature of this study limits the drawing of inferences about causation. Importantly, the potential for reverse causation (also called reverse causality), which refers to a direction of cause-and-effect that is the opposite of the expected outcome, could be a challenge in determining a causal relationship between dietary patterns and health outcome indicators. This tends to be inevitable in observational studies [15]. In this study, a possible direction of causation would include that closer adherence to TDGs results in better health outcome indicators. On the other hand, reverse causality may occur with changes in peoples’ dietary patterns following disease occurrence. For example, people become concerned about being at high risk of having diabetes after being told of such by their general practitioner; thus, they are more likely than the healthier, low-risk population to reduce their intake of foods high in saturated fat and carbohydrates. Thus, although their current diets are now lower in saturated fat and carbohydrates, their risk of developing diabetes may still be high. Thus, to minimize the likelihood of reverse causation as much as possible using the available J-NHNS data, we excluded those who might be aware (or ought to be aware) of their health risks (see the methods section). Other underlying diseases known to be associated with dietary risks (e.g. liver disease, chronic kidney disease, and gastrointestinal disease), may still cause reverse causality.

Fifth, we made a number of comparisons, and some statistically significant associations may have occurred owing to chance events. In this study, the regression approach theoretically assumed the independence of the set of participants between regression models (for the different outcomes). Of course, these models were built with identical participants and hence, this assumption was not fully met. Therefore, the inflation of the alpha error by multiple comparisons can actually be a problem. For example, we observed increased levels of HbA1c among those the 20–39 and 40–59 years age categories who had a higher adherence score (Table 5). This was not concordant with our expected direction of association. A two-way ANOVA could be an alternative analysis approach. However, this approach has a different type of problems. While it models the ‘mean’ variance structure of factors (i.e., independent variables), we would like to focus on 1) the local structure around the quantiles of the adherence score distribution conditioned by the random intercept for the household, and 2) the estimated coefficients of the score on the outcomes (i.e., the direction, magnitude, and significance of the score). A two-way ANOVA cannot explicitly model these two points. Therefore, we decided to use a quantile regression approach instead of two-way ANOVA.

Sixth, the decision to choose HbA1c, SBP, DBP, HDL-C, and BMI as health outcome indicators (the dependent variables) was based on data availability. Future studies should explore outcomes as a whole to attempt to comprehensively evaluate the health impact of TDGs.

Finally, as a consequence of this exclusion of people who have disease risks, the potential impact of poor TDGs adherence on the health outcome indicators might be reduced or eliminated because of a healthier population. However, our sensitivity analyses, i.e. the regression analyses of those who failed to meet the eligibility criteria, demonstrated similar results, indicating that the influence of this limitation may be marginal.

## Supporting information

S1 Table Estimated coefficients of adherence score using quantile/conventional regression among population with poor BMI*. HbA1c: hemoglobin A1c; SBP: systolic blood pressure; DBP: diastolic blood pressure; HDL-C: high-density lipoprotein-cholesterol; BMI: body mass index; CI: Confidence interval; adjusted for sex, occupation, smoking status, alcohol consumption status, and total energy intake as restricted cubic spline; * poor: <18.5 for 18–49 years old, <20.0 for 50–69 years old, <21.5 for 70 years old or more. The 'mean' indicates the parameter estimates of the conventional linear regression, while '25%', '50%', and '75%' show the parameter estimates of the quantile regressions at the 25th, 50th, and 75th percentiles of the adherence score distribution, respectively.
(DOCX)

S2 Table Estimated coefficients of adherence score using quantile/conventional regression among population with proper BMI*. HbA1c: hemoglobin A1c; SBP: systolic blood pressure; DBP: diastolic blood pressure; HDL-C: high-density lipoprotein-cholesterol; BMI: body mass index; CI: Confidence interval; adjusted for sex, occupation, smoking status, alcohol consumption status, and total energy intake as restricted cubic spline; * proper: 18.5–24.9 for 18–49 years old, 20.0–24.9 for 50–69 years old, 21.5–24.9 for 70 years old or more. The 'mean' indicates the parameter estimates of the conventional linear regression, while '25%', '50%', and '75%' show the parameter estimates of the quantile regressions at the 25th, 50th, and 75th percentiles of the adherence score distribution, respectively.
(DOCX)

S3 Table **Estimated coefficients of adherence score using quantile/conventional regression with no eligibility criteria for participants: (A) for score quantile of 25% and 50%, (B) for 75% and mean.** HbA1c: hemoglobin A1c; SBP: systolic blood pressure; DBP: diastolic blood pressure; HDL-C: high-density lipoprotein-cholesterol; BMI: body mass index; CI: Confidence interval; adjusted for sex, occupation, smoking status, alcohol consumption status, and total energy intake as restricted cubic spline. The 'mean' indicates the parameter estimates of the conventional linear regression, while '25%', '50%', and '75%' show the parameter estimates of the quantile regressions at the 25th, 50th, and 75th percentiles of the adherence score distribution, respectively.
(DOCX)
