# Supplementary material for: Does Japan’s national nutrient-based dietary guideline improve lifestyle-related disease outcomes? A retrospective observational cross-sectional study
Source: PLoS One. 2019 Oct 17;14(10):e0224042. doi: 10.1371/journal.pone.0224042 (PMC6797207; doi:10.1371/journal.pone.0224042)
Supplement: S1 Table — HbA1c: hemoglobin A1c; SBP: systolic blood pressure; DBP: diastolic blood pressure; HDL-C: high-density lipoprotein-cholesterol; BMI: body mass index; CI: Confidence interval; adjusted for sex, occupation, smoking status, alcohol consumption status, and total energy intake as restricted cubic spline; * poor: <18.5 for 18–49 years old, <20.0 for 50–69 years old, <21.5 for 70 years old or more. The 'mean' indicates the parameter estimates of the conventional linear regression, while '25%', '50%', and '75%' show the parameter estimates of the quantile regressions at the 25th, 50th, and 75th percentiles of the adherence score distribution, respectively. (DOCX) [file pone.0224042.s001.docx]

**S1 Table. Estimated coefficients of adherence score using quantile/conventional regression among population with poor BMI*.**

| (A) |  |  |  |  |  |
| --- | --- | --- | --- | --- | --- |
| Score quantile | 25% | | 50% (median) | | |
| Age category | Coefficient (95% CI) | p-value | Coefficient (95% CI) | p-value |  |
| HbA1c [%] |  |  |  |  |  |
| 20–39 | 0.007 (-0.003 to 0.016) | 0.190 | -0.002 (-0.014 to 0.011) | 0.810 |  |
| 40–59 | 0.000 (-0.009 to 0.009) | 1.000 | 0.002 (-0.008 to 0.012) | 0.746 |  |
| ≥60 | 0.000 (-0.005 to 0.005) | 0.950 | 0.002 (-0.003 to 0.007) | 0.390 |  |
| SBP [mmHg] |  |  |  |  |  |
| 20–39 | 0.374 (-0.032 to 0.780) | 0.073 | 0.310 (0.014 to 0.606) | <0.05 |  |
| 40–59 | 0.079 (-0.297 to 0.456) | 0.680 | 0.062 (-0.265 to 0.389) | 0.712 |  |
| ≥60 | 0.000 (-0.189 to 0.189) | 1.000 | 0.118 (-0.068 to 0.305) | 0.209 |  |
| DBP [mmHg] |  |  |  |  |  |
| 20–39 | 0.120 (-0.160 to 0.400) | 0.403 | 0.181 (-0.035 to 0.396) | 0.103 |  |
| 40–59 | 0.247 (-0.010 to 0.505) | 0.061 | -0.017 (-0.301 to 0.267) | 0.908 |  |
| ≥60 | -0.018 (-0.175 to 0.139) | 0.819 | 0.061 (-0.093 to 0.214) | 0.433 |  |
| HDL-C [mg/dL] |  |  |  |  |  |
| 20–39 | 0.204 (-0.271 to 0.679) | 0.401 | 0.197 (-0.351 to 0.745) | 0.482 |  |
| 40–59 | -0.066 (-0.513 to 0.381) | 0.773 | -0.273 (-0.666 to 0.12) | 0.175 |  |
| ≥60 | -0.163 (-0.465 to 0.138) | 0.282 | -0.008 (-0.310 to 0.294) | 0.958 |  |
| BMI |  |  |  |  |  |
| 20–39 | 0.006 (-0.032 to 0.044) | 0.748 | -0.001 (-0.030 to 0.028) | 0.930 |  |
| 40–59 | -0.002 (-0.037 to 0.033) | 0.899 | 0.015 (-0.014 to 0.044) | 0.310 |  |
| ≥60 | 0.027 (-0.005 to 0.058) | 0.096 | 0.038 (0.005 to 0.070) | <0.05 |  |
| (B) |  |  |  |  |  |
| Score quantile | 75% | | Mean | | |
| Age category | Coefficient (95% CI) | p-value | Coefficient (95% CI) | p-value |  |
| HbA1c [%] |  |  |  |  |  |
| 20–39 | -0.004 (-0.019 to 0.011) | 0.608 | -0.003 (-0.010 to 0.003) | 0.330 |  |
| 40–59 | 0.000 (-0.009 to 0.009) | 1.000 | 0.002 (-0.004 to 0.008) | 0.553 |  |
| ≥60 | 0.004 (0.000 to 0.009) | 0.063 | 0.003 (-0.003 to 0.009) | 0.326 |  |
| SBP [mmHg] |  |  |  |  |  |
| 20–39 | 0.270 (-0.142 to 0.683) | 0.201 | 0.304 (0.064 to 0.543) | <0.05 |  |
| 40–59 | -0.211 (-0.585 to 0.163) | 0.269 | -0.019 (-0.255 to 0.218) | 0.878 |  |
| ≥60 | 0.233 (0.041 to 0.425) | <0.05 | 0.100 (-0.120 to 0.320) | 0.372 |  |
| DBP [mmHg] |  |  |  |  |  |
| 20–39 | 0.133 (-0.269 to 0.536) | 0.517 | 0.125 (-0.072 to 0.322) | 0.214 |  |
| 40–59 | -0.142 (-0.365 to 0.081) | 0.213 | 0.060 (-0.112 to 0.231) | 0.495 |  |
| ≥60 | 0.138 (-0.016 to 0.292) | 0.078 | 0.055 (-0.100 to 0.210) | 0.490 |  |
| HDL-C [mg/dL] |  |  |  |  |  |
| 20–39 | -0.124 (-0.702 to 0.455) | 0.676 | 0.015 (-0.300 to 0.330) | 0.925 |  |
| 40–59 | -0.096 (-0.66 to 0.469) | 0.740 | -0.298 (-0.626 to 0.03) | 0.076 |  |
| ≥60 | 0.142 (-0.163 to 0.447) | 0.355 | 0.012 (-0.289 to 0.314) | 0.936 |  |
| BMI |  |  |  |  |  |
| 20–39 | -0.007 (-0.020 to 0.005) | 0.263 | 0.004 (-0.019 to 0.027) | 0.710 |  |
| 40–59 | 0.008 (-0.031 to 0.046) | 0.699 | 0.000 (-0.024 to 0.024) | 0.994 |  |
| ≥60 | 0.047 (0.016 to 0.079) | <0.01 | 0.038 (0.011 to 0.065) | <0.01 |  |

HbA1c: hemoglobin A1c; SBP: systolic blood pressure; DBP: diastolic blood pressure; HDL-C: high-density lipoprotein-cholesterol; BMI: body mass index; CI: Confidence interval; adjusted for sex, occupation, smoking status, alcohol consumption status, and total energy intake as restricted cubic spline; * poor: <18.5 for 18–49 years old, <20.0 for 50–69 years old, <21.5 for 70 years old or more. The 'mean' indicates the parameter estimates of the conventional linear regression, while '25%', '50%', and '75%' show the parameter estimates of the quantile regressions at the 25th, 50th, and 75th percentiles of the adherence score distribution, respectively.
