# Supplementary material for: Does Japan’s national nutrient-based dietary guideline improve lifestyle-related disease outcomes? A retrospective observational cross-sectional study
Source: PLoS One. 2019 Oct 17;14(10):e0224042. doi: 10.1371/journal.pone.0224042 (PMC6797207; doi:10.1371/journal.pone.0224042)
Supplement: S2 Table — HbA1c: hemoglobin A1c; SBP: systolic blood pressure; DBP: diastolic blood pressure; HDL-C: high-density lipoprotein-cholesterol; BMI: body mass index; CI: Confidence interval; adjusted for sex, occupation, smoking status, alcohol consumption status, and total energy intake as restricted cubic spline; * proper: 18.5–24.9 for 18–49 years old, 20.0–24.9 for 50–69 years old, 21.5–24.9 for 70 years old or more. The 'mean' indicates the parameter estimates of the conventional linear regression, while '25%', '50%', and '75%' show the parameter estimates of the quantile regressions at the 25th, 50th, and 75th percentiles of the adherence score distribution, respectively. (DOCX) [file pone.0224042.s002.docx]

**S2 Table. Estimated coefficients of adherence score using quantile/conventional regression among population with proper BMI*.**

| (A) |  |  |  | |  |
| --- | --- | --- | --- | --- | --- |
| Score quantile | 25% | | | 50% (median) | |
| Age category | Coefficient (95% CI) | p-value | Coefficient (95% CI) | | p-value |
| HbA1c [%] |  |  |  | |  |
| 20–39 | 0.002 (-0.001 to 0.004) | 0.196 | 0.004 (0.002 to 0.007) | | <0.01 |
| 40–59 | 0.003 (0.000 to 0.005) | 0.073 | 0.005 (0.001 to 0.008) | | <0.05 |
| ≥60 | 0.000 (-0.005 to 0.005) | 0.948 | 0.002 (-0.003 to 0.007) | | 0.400 |
| SBP [mmHg] |  |  |  | |  |
| 20–39 | -0.080 (-0.176 to 0.015) | 0.097 | 0.000 (-0.095 to 0.096) | | 0.993 |
| 40–59 | -0.079 (-0.194 to 0.035) | 0.171 | 0.012 (-0.107 to 0.132) | | 0.837 |
| ≥60 | -0.011 (-0.174 to 0.151) | 0.891 | 0.087 (-0.069 to 0.243) | | 0.268 |
| DBP [mmHg] |  |  |  | |  |
| 20–39 | -0.036 (-0.123 to 0.052) | 0.418 | 0.054 (-0.036 to 0.143) | | 0.235 |
| 40–59 | -0.012 (-0.100 to 0.076) | 0.779 | 0.051 (-0.037 to 0.138) | | 0.250 |
| ≥60 | -0.132 (-0.231 to -0.033) | <0.01 | -0.035 (-0.138 to 0.067) | | 0.494 |
| HDL-C [mg/dL] |  |  |  | |  |
| 20–39 | -0.213 (-0.339 to -0.086) | <0.01 | -0.083 (-0.21 to 0.044) | | 0.194 |
| 40–59 | -0.247 (-0.405 to -0.09) | <0.01 | -0.128 (-0.29 to 0.033) | | 0.117 |
| ≥60 | -0.206 (-0.393 to -0.02) | <0.05 | -0.079 (-0.269 to 0.111) | | 0.406 |
| BMI |  |  |  | |  |
| 20–39 | -0.006 (-0.025 to 0.013) | 0.519 | 0.006 (-0.012 to 0.024) | | 0.491 |
| 40–59 | -0.013 (-0.029 to 0.003) | 0.117 | 0.002 (-0.014 to 0.018) | | 0.801 |
| ≥60 | -0.007 (-0.024 to 0.011) | 0.446 | 0.004 (-0.013 to 0.022) | | 0.625 |
| (B) |  |  |  | |  |
| Score quantile | 75% | | | Mean | |
| Age category | Coefficient (95% CI) | p-value | Coefficient (95% CI) | | p-value |
| HbA1c [%] |  |  |  | |  |
| 20–39 | 0.007 (0.004 to 0.009) | <0.001 | 0.004 (0.001 to 0.007) | | <0.01 |
| 40–59 | 0.007 (0.004 to 0.01) | <0.001 | 0.005 (0.002 to 0.008) | | <0.01 |
| ≥60 | 0.004 (0.000 to 0.009) | 0.071 | 0.002 (-0.003 to 0.007) | | 0.368 |
| SBP [mmHg] |  |  |  | |  |
| 20–39 | 0.087 (-0.004 to 0.178) | 0.060 | -0.003 (-0.107 to 0.100) | | 0.952 |
| 40–59 | 0.084 (-0.038 to 0.207) | 0.172 | 0.016 (-0.092 to 0.125) | | 0.768 |
| ≥60 | 0.196 (0.039 to 0.353) | <0.05 | 0.070 (-0.081 to 0.220) | | 0.366 |
| DBP [mmHg] |  |  |  | |  |
| 20–39 | 0.132 (0.042 to 0.223) | <0.01 | 0.055 (-0.032 to 0.141) | | 0.217 |
| 40–59 | 0.128 (0.041 to 0.215) | <0.01 | 0.041 (-0.035 to 0.117) | | 0.287 |
| ≥60 | 0.037 (-0.063 to 0.138) | 0.457 | -0.044 (-0.158 to 0.071) | | 0.453 |
| HDL-C [mg/dL] |  |  |  | |  |
| 20–39 | 0.079 (-0.052 to 0.209) | 0.231 | -0.055 (-0.194 to 0.084) | | 0.437 |
| 40–59 | 0.030 (-0.132 to 0.191) | 0.715 | -0.111 (-0.255 to 0.033) | | 0.133 |
| ≥60 | 0.083 (-0.117 to 0.282) | 0.409 | -0.050 (-0.256 to 0.156) | | 0.635 |
| BMI |  |  |  | |  |
| 20–39 | 0.024 (0.005 to 0.043) | <0.05 | 0.010 (-0.008 to 0.028) | | 0.300 |
| 40–59 | 0.016 (0.000 to 0.032) | <0.05 | 0.001 (-0.015 to 0.018) | | 0.862 |
| ≥60 | 0.014 (-0.004 to 0.032) | 0.125 | 0.005 (-0.015 to 0.024) | | 0.644 |

HbA1c: hemoglobin A1c; SBP: systolic blood pressure; DBP: diastolic blood pressure; HDL-C: high-density lipoprotein-cholesterol; BMI: body mass index; CI: Confidence interval; adjusted for sex, occupation, smoking status, alcohol consumption status, and total energy intake as restricted cubic spline; * proper: 18.5­–24.9 for 18–49 years old, 20.0–24.9 for 50–69 years old, 21.5–24.9 for 70 years old or more. The 'mean' indicates the parameter estimates of the conventional linear regression, while '25%', '50%', and '75%' show the parameter estimates of the quantile regressions at the 25th, 50th, and 75th percentiles of the adherence score distribution, respectively.
