# Supplementary material for: Does Japan’s national nutrient-based dietary guideline improve lifestyle-related disease outcomes? A retrospective observational cross-sectional study
Source: PLoS One. 2019 Oct 17;14(10):e0224042. doi: 10.1371/journal.pone.0224042 (PMC6797207; doi:10.1371/journal.pone.0224042)
Supplement: S3 Table — Estimated coefficients of adherence score using quantile/conventional regression with no eligibility criteria for participants: (A) for score quantile of 25% and 50%, (B) for 75% and mean. HbA1c: hemoglobin A1c; SBP: systolic blood pressure; DBP: diastolic blood pressure; HDL-C: high-density lipoprotein-cholesterol; BMI: body mass index; CI: Confidence interval; adjusted for sex, occupation, smoking status, alcohol consumption status, and total energy intake as restricted cubic spline. The 'mean' indicates the parameter estimates of the conventional linear regression, while '25%', '50%', and '75%' show the parameter estimates of the quantile regressions at the 25th, 50th, and 75th percentiles of the adherence score distribution, respectively. (DOCX) [file pone.0224042.s003.docx]

**S3 Table. Estimated coefficients of adherence score using quantile/conventional regression** **with no eligibility criteria for participants: (A) for score quantile of 25% and 50%, (B) for 75% and mean.**

| (A) |  |  |  |  |
| --- | --- | --- | --- | --- |
| Score quantile | 25% | | 50% (median) | |
| Age category | Coefficient (95% CI) | p-value | Coefficient (95% CI) | p-value |
| HbA1c [%] |  |  |  |  |
| 20–39 | 0.000 (-0.003 to 0.002) | 0.691 | 0.002 (0.000 to 0.004) | 0.100 |
| 40–59 | -0.005 (-0.009 to -0.002) | <0.01 | -0.003 (-0.007 to 0.001) | 0.119 |
| ≥60 | -0.008 (-0.012 to -0.004) | <0.01 | -0.002 (-0.007 to 0.002) | 0.233 |
| SBP [mmHg] |  |  |  |  |
| 20–39 | -0.087 (-0.199 to 0.024) | 0.122 | 0.030 (-0.084 to 0.143) | 0.603 |
| 40–59 | -0.278 (-0.384 to -0.172) | <0.001 | -0.106 (-0.211 to -0.001) | <0.05 |
| ≥60 | -0.244 (-0.336 to -0.153) | <0.001 | -0.087 (-0.176 to 0.002) | 0.054 |
| DBP [mmHg] |  |  |  |  |
| 20–39 | -0.052 (-0.147 to 0.043) | 0.275 | 0.054 (-0.043 to 0.151) | 0.269 |
| 40–59 | -0.134 (-0.212 to -0.057) | <0.01 | -0.025 (-0.102 to 0.052) | 0.517 |
| ≥60 | -0.156 (-0.222 to -0.090) | <0.001 | -0.068 (-0.134 to -0.003) | <0.05 |
| HDL-C [mg/dL] |  |  |  |  |
| 20–39 | -0.187 (-0.308 to -0.065) | <0.01 | -0.044 (-0.167 to 0.080) | 0.479 |
| 40–59 | -0.164 (-0.257 to -0.070) | <0.01 | -0.020 (-0.115 to 0.075) | 0.671 |
| ≥60 | -0.189 (-0.291 to -0.087) | <0.01 | -0.048 (-0.147 to 0.051) | 0.334 |
| BMI |  |  |  |  |
| 20–39 | -0.030 (-0.059 to -0.001) | <0.05 | -0.001 (-0.030 to 0.028) | 0.960 |
| 40–59 | -0.075 (-0.103 to -0.047) | <0.001 | -0.043 (-0.071 to -0.016) | <0.01 |
| ≥60 | -0.071 (-0.089 to -0.053) | <0.001 | -0.045 (-0.063 to -0.027) | <0.001 |
| (B) |  |  |  |  |
| Score quantile | 75% | | Mean | |
| Age category | Coefficient (95% CI) | p-value | Coefficient (95% CI) | p-value |
| HbA1c [%] |  |  |  |  |
| 20–39 | 0.005 (0.002 to 0.007) | <0.001 | 0.002 (-0.001 to 0.005) | 0.168 |
| 40–59 | 0.000 (-0.004 to 0.004) | 0.939 | -0.001 (-0.005 to 0.002) | 0.494 |
| ≥60 | 0.005 (-0.001 to 0.010) | 0.104 | 0.000 (-0.004 to 0.003) | 0.903 |
| SBP [mmHg] |  |  |  |  |
| 20–39 | 0.162 (0.046 to 0.278) | <0.01 | 0.045 (-0.057 to 0.148) | 0.386 |
| 40–59 | 0.031 (-0.073 to 0.134) | 0.556 | -0.084 (-0.191 to 0.024) | 0.126 |
| ≥60 | 0.055 (-0.039 to 0.150) | 0.246 | -0.071 (-0.159 to 0.017) | 0.116 |
| DBP [mmHg] |  |  |  |  |
| 20–39 | 0.167 (0.069 to 0.265) | <0.01 | 0.063 (-0.019 to 0.145) | 0.135 |
| 40–59 | 0.073 (-0.003 to 0.150) | 0.060 | -0.016 (-0.087 to 0.055) | 0.657 |
| ≥60 | 0.027 (-0.039 to 0.094) | 0.415 | -0.056 (-0.112 to -0.001) | <0.05 |
| HDL-C [mg/dL] |  |  |  |  |
| 20–39 | 0.124 (0.000 to 0.249) | 0.050 | -0.019 (-0.137 to 0.100) | 0.759 |
| 40–59 | 0.152 (0.054 to 0.250) | <0.01 | -0.006 (-0.106 to 0.095) | 0.911 |
| ≥60 | 0.115 (0.013 to 0.218) | 0.027 | -0.021 (-0.102 to 0.059) | 0.604 |
| BMI |  |  |  |  |
| 20–39 | 0.040 (0.009 to 0.071) | <0.05 | 0.008 (-0.025 to 0.040) | 0.645 |
| 40–59 | -0.007 (-0.035 to 0.022) | 0.637 | -0.033 (-0.057 to -0.008) | <0.01 |
| ≥60 | -0.016 (-0.034 to 0.003) | 0.100 | -0.041 (-0.059 to -0.023) | <0.001 |

HbA1c: hemoglobin A1c; SBP: systolic blood pressure; DBP: diastolic blood pressure; HDL-C: high-density lipoprotein-cholesterol; BMI: body mass index; CI: Confidence interval; adjusted for sex, occupation, smoking status, alcohol consumption status, and total energy intake as restricted cubic spline. The 'mean' indicates the parameter estimates of the conventional linear regression, while '25%', '50%', and '75%' show the parameter estimates of the quantile regressions at the 25th, 50th, and 75th percentiles of the adherence score distribution, respectively.
